# Integrating network pharmacology and experimental verification to decipher the immunomodulatory effect of Bu-Zhong-Yi-Qi-Tang against poly (I:C)-induced pulmonary inflammation

**DOI:** 10.3389/fphar.2022.1015486

**Published:** 2022-10-11

**Authors:** Liufang Hu, Marina Yamamoto, Jiali Chen, Huifang Duan, Jing Du, Liangliang He, Danfeng Shi, Xinsheng Yao, Takayuki Nagai, Hiroaki Kiyohara, Zhihong Yao

**Affiliations:** ^1^ College of Pharmacy, Institute of Traditional Chinese Medicine and Natural Products, Jinan University, Guangzhou, China; ^2^ Laboratory of Biochemical Pharmacology for Phytomedicines, Ōmura Satoshi Memorial Institute and Graduate School of Infection Control Sciences, Kitasato University, Tokyo, Japan; ^3^ Tong Ren Tang Technologies Co. Ltd, Beijing, China; ^4^ International Cooperative Laboratory of Traditional Chinese Medicine Modernization and Innovative Drug Development of Ministry of Education (MOE) of China, Jinan University, Guangzhou, China; ^5^ Oriental Medicine Research Center, Kitasato University, Tokyo, Japan

**Keywords:** Bu-zhong-yi-qi-tang, traditional Chinese medicine formula, pulmonary inflammation, immunomodulatory effect, network pharmacology, leukocyte transendothelial migration

## Abstract

Pulmonary inflammation caused by respiratory tract viral infections is usually associated with acute exacerbation of respiratory diseases, such as asthma and chronic obstructive pulmonary disease (COPD). Therefore, maintaining the pulmonary immune homeostasis is particular important for prevention of the acute exacerbation. Bu-Zhong-Yi-Qi-Tang (BZYQT), a traditional Chinese medicine formula, has been broadly used to improve respiratory and gastrointestinal disorders in China for over 700 years. Previously, we have found the regulatory activity of BZYQT on the lower respiratory immune system, while its potential effects during pulmonary inflammation remain unknown. Thus, the current study focused on deciphering its immunomodulatory effect and potential mechanism against pulmonary inflammation by using a viral RNA analogue, poly (I:C), induced murine pulmonary inflammation model and BEAS-2B cell model coupled with network pharmacology. Inflammatory cells in the bronchoalveolar lavage fluid were counted through microscope examination according to the cell’s morphology and staining characteristics; protein and gene levels of inflammatory mediators were determined with Elisa and quantitative PCR, respectively; network pharmacology was conducted based on 46 BZYQT-related potential bioactive components, pulmonary inflammation and immune-related targets. Our results indicated that the recruitment of neutrophils and the expression of *Adgre1* (encoding the F4/80, which is a macrophage marker) in the lung induced by poly (I:C) were significantly reduced after BZYQT treatment, and these effects were further demonstrated to be related to the interference of leukocyte transendothelial migration from the decreased levels of CXCL10, IL-6, TNF-α, CXCL2, ICAM-1, VCAM-1, and E/P-selectins. Furthermore, BZYQT inhibited the CXCL10, TNF-α, and IFN-β expression of poly (I:C)-challenged BEAS-2B cells in a dose-dependent manner. Through integrating results from network pharmacology, experiments, and the published literature, isoliquiritigenin, *Z*-ligustilide, atractylenolide I, atractylenolide III, formononetin, ferulic acid, hesperidin, and cimigenoside were presumed as the bioactive components of BZYQT against pulmonary inflammation. Overall, our findings demonstrated that BZYQT possesses a pronounced immunomodulatory effect on poly (I:C)-induced pulmonary inflammation, which provides a pharmacological basis for BZYQT in the treatment of respiratory disorders.

## Graphical Abstract

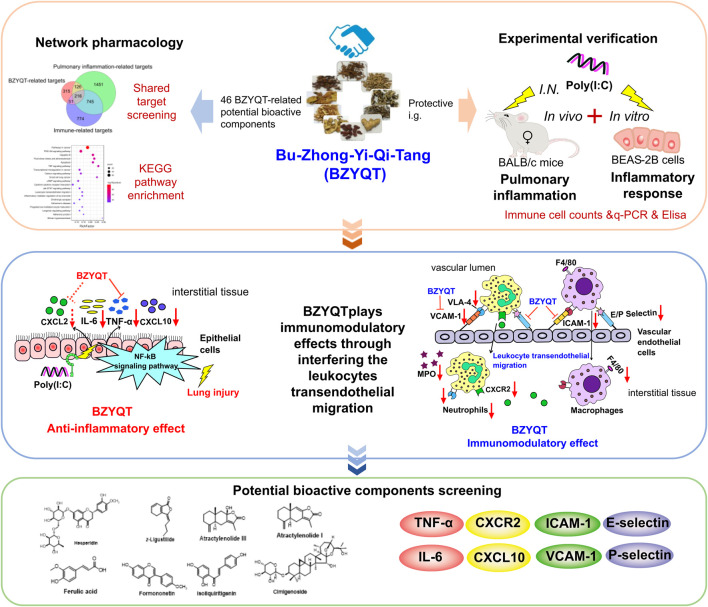

## 1 Introduction

The mucosal surface of the respiratory system is extremely susceptible to various viral and bacterial infections due to direct exposure to the external environment. As a result, to prevent infections and preserve pulmonary homeostasis, the respiratory tract must orchestrate a series of immune responses (Allie and Randall, 2017; Wiesner and Klein, 2017). For example, inflammatory response, a process in which a large number of inflammatory mediators released by activated macrophages act to recruit cells into the infected tissues is believed to help destroy the pathogen. However, excessive inflammatory responses in the airway caused by viral infections usually induce acute exacerbations of respiratory diseases, such as chronic obstructive pulmonary disease (COPD) and asthma, and accelerate lung function decline subsequently, which becomes the major cause of morbidity and mortality (Stowell et al., 2009; Harris et al., 2013). Moreover, it was proposed that impaired immune regulation probably played a role in COPD (Rabe and Watz, 2017). Therefore, it is necessary to seek out potential therapies to balance the immune response, which may serve to alleviate exacerbations and improve pulmonary immunity.

Bu-Zhong-Yi-Qi-Tang (BZYQT), a well-known traditional Chinese medicine (TCM) formula recorded in the “Treatise on Spleen and Stomach (Pi-Wei-Lun, 1249 A.D.)” for invigorating spleen and replenishing qi (a TCM term, which is thought to provide the vital energy of the body by TCM), has been extensively used in clinical practice of China to improve respiratory and gastrointestinal disorders (Zheng et al., 2014; Kim et al., 2017; Liu et al., 2019). It is composed of ten herbal medicines, including *Astragali Radix* praeparata cum melle, wheat-fried *Atractylodis Macrocephalae Rhizoma*, *Codonopsis Radix*, *Glycyrrhizae Radix et Rhizoma* praeparata cum melle, *Citri Reticulatae Pericarpium*, *Angelicae Sinensis Radix*, *Cimicifugae Rhizoma*, *Bupleuri Radix*, *Jujubae Fructus*, *Zingiberis Rhizoma Recens*. In addition to having a profound influence in China, BZYQT is also prevalent in Japan (named hochuekkito) and South Korea (called Bojungikki-tang) due to its extensive pharmacological activities like immunomodulation, anti-inflammation, anti-fatigue, anti-bacterial, etc. (Yamaoka et al., 2000; Yang and Yu, 2008; Chen et al., 2009; Yang et al., 2015; Liu et al., 2019). Currently, BZYQT has been reported with multiple effects on respiratory diseases, for example, reducing systemic inflammation, improving the exercise capacity, lung function, and quality of life for COPD patients (Shinozuka et al., 2007; Chen et al., 2016); decreasing the recurrent attack rate of asthma and allergic rhinitis (Yang and Yu, 2008); inhibiting the rhinovirus infection in human tracheal epithelial cells (Yamaya et al., 2007). Our previous study demonstrated that BZYQT could negatively regulate the pulmonary immune system from the aspect of antigen-specific antibody responses (Liu et al., 2019). However, the potential effects of BZYQT on pulmonary inflammation are still unclear.

The efficacy of BZYQT on diseases is thought to be linked with its multi-component, multi-target, and multi-pathway. Previously, a total of 161 compounds and 162 xenobiotics have been characterized in BZYQT water extracts and BZYQT-related bio-samples, respectively, including flavonoids, triterpenoids, lactone, alkaloid, and other structural types (Hu et al., 2019; Liu et al., 2019). Therefore, there remain difficulties to understand the bioactive components and the underlying molecular mechanisms of BZYQT in such a complex system. In recent years, network pharmacology has gained more popularity as a comprehensive and efficient technique for establishing a strong link between TCM compounds and targets of a complex biological system, which helps to decipher the active components and the molecular mechanism of TCM. For instance, possible action mechanisms as well as the bioactive ingredients of Xin-Sheng-Hua Granule, and the potential targets of *Radix Curcumae* were explained by using network pharmacology (Tao et al., 2013; Pang et al., 2018).

Given these facts, the present study focused on the immunomodulatory effect of BZYQT in pulmonary acute exacerbation by integrating the network pharmacology approach with experimental verification. First, the murine pulmonary inflammation model and airway epithelial cell inflammatory response model induced by a viral RNA analogue, poly (I:C), were used to evaluate the immunomodulatory effect of BZYQT. Then, the probable mechanism of BZYQT for alleviating pulmonary inflammation was explored and validated through integrating network pharmacology of BZYQT-related bioavailable components with experiments. At last, its bioactive components against pulmonary inflammation were screened out according to the relationship of “compounds” and “targets,” and the literature. This study would help to promote the clinical application of BZYQT and provide meaningful guidance for further understanding of its potential molecular mechanism.

## 2 Materials and methods

### 2.1 Reagents and materials

**FIGURE 1:**
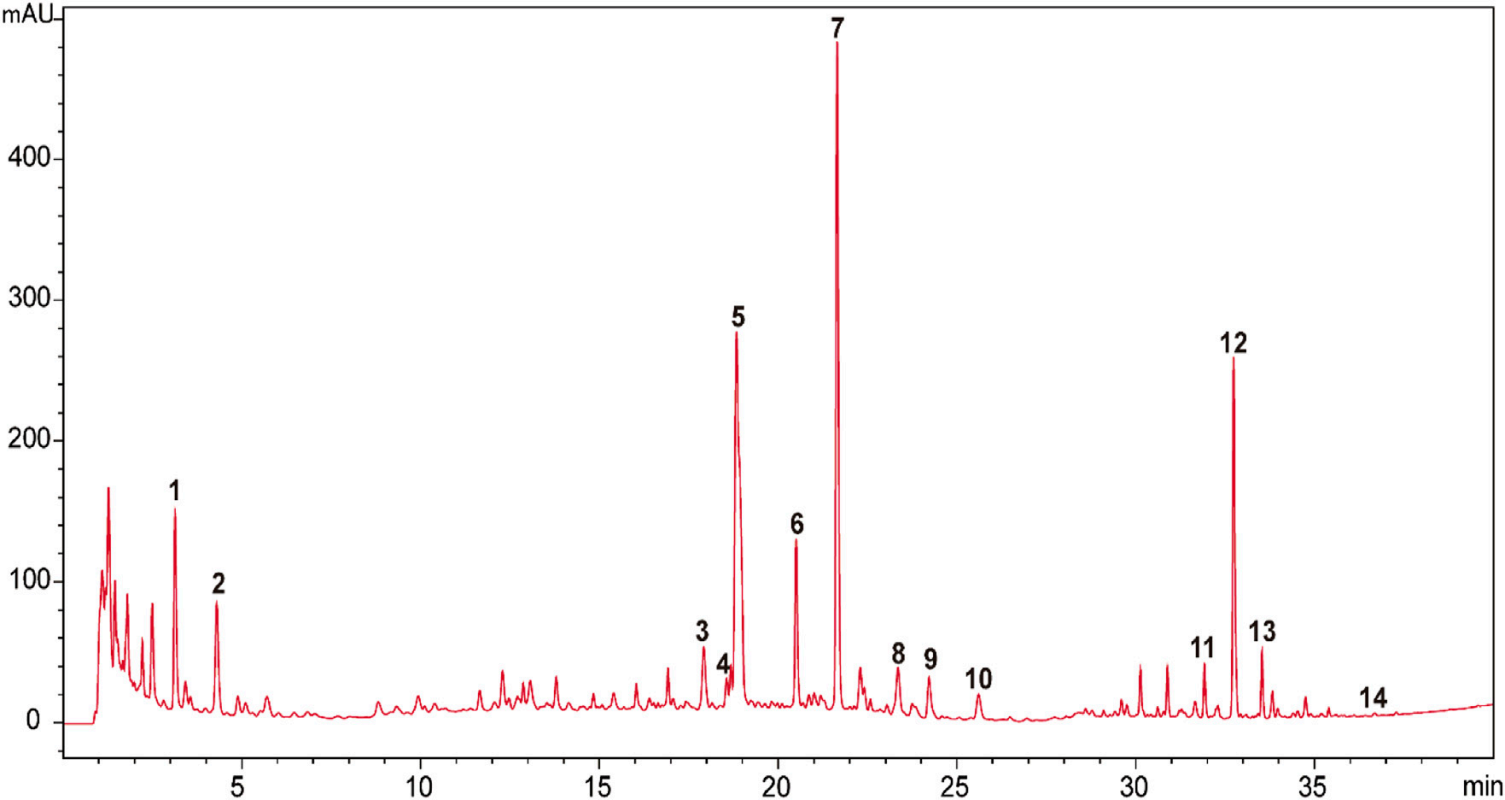
HPLC-UVD fingerprint of BZYQT. **1.** (3R)-2,3-dihydro-3,5-dihydroxy-6-methyl-4(4H)-pranone; **2.**5-hydroxymethylfurfural; **3.** ferulic acid; **4.** neoliquiritin; **5.** liquiritin; **6.** narirutin; **7.** hesperidin; **8.** lobetyolin; **9.** ononin; **10.** cimicifugic acid F; **11.** formononetin; **12.**18*β*-glycyrrhizic acid; **13.** uralsaponin B; **14.** Z-ligustilide. Analysis conditions: Agilent 1200 HPLC equipped with a UVD detector was used as an analytic system. Column and temperature: Poroshell 120 EC-C18 (4.6 mm × 100 mm, 2.7 μm), 40°C; mobile phases: water (A) and acetonitrile (B) (both containing 0.1% formic acid); flow rate: 1 ml/min; injection volume: 5 μl; detection wavelength: 280 nm (0–24 min) and 254 (24–40 min); elution program as follows: 0–5 min (2%), 5–10 min (2%–8.5%), 10–15 min (8.5%–15%), 15–23 min (15%–23%), 23–25 min (23%–25%), 25–30 min (25%–40%), 30–35 min (40%–60%), 35–40 min (60%–100%). Compounds were identified by comparing with reference standards. Preliminary quantitative analysis showed that the contents of hesperidin (Peak 7), 18*β*-glycyrrhizic acid (Peak 12), lobetyolin (Peak 8), ferulic acid (Peak 3), ononin (Peak 9), narirutin (Peak 6) were 3,600, 3,300, 314.7, 166.7, 111.5, 99.2 μg/g, respectively.

The raw herbal medicines of *Astragali Radix* praeparata cum melle (*Astragalus membranaceus* (Fisch.) Bunge, root, honeyed), *Codonopsis Radix* (*Codonopsis pilosula* (Franch.) Nannf., root), wheat-fried *Atractylodis Macrocephalae Rhizoma* (*Atractylis macrocephala* (Koidz.), rhizome, wheat-fried), *Glycyrrhizae Radix et Rhizoma* praeparata cum melle (*Glycyrrhiza uralensis* Fisch., root and rhizome, honeyed), *Bupleuri Radix* (*Bupleurum chinense* DC., root)*, Angelicae Sinensis Radix* (*Angelica sinensis* (Oliv.) Diels, root), *Citrus Reticulatae Pericarpium* (*Citrus reticulata* Blanco, pericarp), *Cimicifugae Rhizoma* (*Cimicifuga heracleifolia* Kom., rhizome), *Zingiberis Rhizoma Recens* (*Zingiber officinale* Roscoe, rhizome), and *Jujubae Fructus* (*Ziziphus jujuba* Mill., fruit) were generously provided by Tong Ren Tang Technologies Co. Ltd. (Beijing, China). BZYQT water extracts were basically prepared in the same way as our previous research (Liu et al., 2019), but the extracts were freeze-dried (average yield: 38.80 ± 0.69). HPLC-UVD fingerprint analysis of BZYQT was carried out for quality control (Figure 1). Poly (I:C) (high molecular weight) was purchased from InvivoGen (San Diego, CA, United States).

### 2.2 Animal experiments

#### 2.2.1 Ethics

The animal experiments were approved by the Institutional Animal Care and Use Committee of Kitasato University (approval No. 19–003) and were carried out following the Regulations for Care and Use of Laboratory Animals in Kitasato University and the Guidelines for Proper Conduct of Animal Experiments from the Science Council of Japan.

#### 2.2.2 Animals

Specific pathogen-free BALB/c mice (6 weeks old, female) were purchased from Japan SLC, Inc. (Hamamatsu, Japan). All mice were fed with a standard alfalfa-free laboratory diet (PicoLab^®^ Mouse Diet 20 5058, Lab Supply, Fort Worth, TX, United States) and water *ad libitum* in a standard laboratory environment (humidity: 55 ± 10%, temperature: 23 ± 2°C, 12 h light/dark cycle). Mice would acclimate for 14 days before experiments.

#### 2.2.3 Poly (I:C)-induced murine pulmonary inflammation model and drug administration

The poly (I:C)-induced murine pulmonary inflammation model was established according to the method in our published research (Hu et al., 2021). Briefly, mice were anesthetized by *i. p.* injection of Somnopentyl^®^ (Kyoritsu Seiyaku Corp., Tokyo, Japan) or a mixture of three kinds of anesthetics [Domitor^®^ (Nippon Zenyaku Kogyo, Kōriyama, Japan), Dormicum^®^ (Maruishi Pharmaceutical, Osaka, Japan), Vetorphale^®^ (Meiji Animal Health, Kumamoto, Japan)] after drug administration and were inoculated with 25 μl saline solution of poly (I:C) (1 mg/ml) *via* the right nostril; the procedures were conducted for three consecutive days. In this study, mice were randomly divided into three groups: normal, model, and BZYQT groups. For protective administration, mice in the BZYQT group were intragastrically given aqueous solutions (0.3 ml/mouse) of BZYQT extracts at a dose of 1.5 g/kg/day before the poly (I:C) inoculation, while normal and model groups were treated with the equal volume of vehicle; for prophylactic administration, BZYQT was administrated prophylactically for additional 7 days before poly (I:C) inoculation. The daily dose of BZYQT was determined according to the previous study (Ideno et al., 2020), which is equivalent to 1.5 folds of the converted clinical dosage (Commission, 2015). Twenty-4 h after the last poly (I:C) intranasal inoculation, mice were sacrificed by inhaling isoflurane for collecting bronchoalveolar lavage fluid (BALF) and lung tissues according to previous research (Kajita et al., 2019; Hu et al., 2021). All samples were stored at −80°C before analysis.

### 2.3 Cell experiments

#### 2.3.1 Cell culture

Normal human bronchial/pulmonary epithelial cell line, BEAS-2B cells (ATCC^®^ CRL-9609™) were purchased from American Type Culture Collection (Manassas, VA, United States). Cryopreserved cell stock grew and expanded in BEGM™ BulletKit™ medium (Lonza Walkersville Inc., Walkersville, MD, United States) at the incubator of 37°C and 5% CO_2_. Cell passage would be done while cells grew to a density of 80%–90%.

#### 2.3.2 Cytotoxicity evaluation of BZYQT

MTT assay was conducted to evaluate the cytotoxicity of BZYQT on BEAS-2B cells. Briefly, BEAS-2B cells were seeded in BioCoat™ Collagen I 96-well Microtest™ Plates (Corning, Franklin Lakes, NJ, United States) at a density of 8×10^4^ cells/mL (100 µl/well). When cells grew to a density of 90%, the medium was replaced by a fresh medium containing 1 μg/ml poly (I:C) and 1, 10, 100, 500, or 1000 μg/ml BZYQT (*n* = 4 in each BZYQT concentration). Wells added with medium only containing poly (I:C) were used as control (*n* = 4). After incubation for 24 h, the original medium was replaced by a 100 µl medium containing 0.5 mg/ml MTT solutions and incubated for 2 h. Later, the supernatants were removed, and the formazan was dissolved in 200 μl DMSO. The absorbance was measured at 535 nm by using Infinite^®^ M200 Microplate Reader (Tecan, Männedorf, Switzerland).

#### 2.3.3 Poly (I:C)-challenged inflammation in BEAS-2B cells

BEAS-2B cells were seeded in BioCoat™ Collagen I 6-well Multi-well Plates (Corning) at a density of 1 × 10^5^ cells/ml (2 ml/well) for poly (I:C) induction. When cells grew to a density of 90%, they were pretreated with fresh medium containing poly (I:C) (1 μg/ml) and BZYQT aqueous solutions (100, 250, or 500 μg/ml) or poly (I:C) and sterile water. For control, 0.5% (*v/v*) sterile water in the medium was added. After 24 h incubation, the cell supernatants were collected, and cells were stored at −80°C until RNA extraction.

### 2.4 White blood cell count in the BALF

Total WBC counts were measured by an automated hematology analyzer (F-820, Sysmex, Tokyo, Japan). Differential cell counts in the BALF were determined by making a smear and using the May-Grünwald-Giemsa staining method described in our published paper (Hu, et al., 2021).

### 2.5 Real-time quantitative PCR

The mRNA expression levels of inflammatory mediators in the lung and BEAS-2B cells were determined through real-time quantitative PCR. Sepasol^®^-RNA I Super G (Nacalai Tesque Inc., Kyoto, Japan), an acid guanidinium thiocyanate-phenol-chloroform extraction reagent, was used to extract total RNA from lung tissue or BEAS-2B cells. Single-strand cDNA was synthesized from 2.0 μg total RNA by reverse transcription using ReverTra Ace^®^ qPCR RT Master Mix with gDNA Remover kits (TOYOBO Co., Ltd., Osaka, Japan). Amplification of cDNA was performed by quantitative PCR, using THUNDERBIRD^®^ SYBR^®^ qPCR Mix (TOYOBO) with specific primers to measure the mRNA expression. Glyceraldehyde-3-phosphate dehydrogenase (murine: *Gapdh,* human: *GAPDH*) was used as a housekeeping gene. The sequences of murine and human primer were listed in Supplementary Tables S1, S2. In this study, relative quantitative standard curve method was employed to calculate the mRNA expression of the target gene. Data were considered reliable when the R2 value was great than 0.99, and reaction efficiency was 90%–110%.

### 2.6 Enzyme-linked immunosorbent assay and myeloperoxidase activity evaluation

The protein content of inflammatory mediators in BALF was measured by DuoSet™ ELISA Kit (R&D Systems, Minneapolis, MN, United States) according to the manufacturer’s manual. MPO activity was evaluated by reacting with TMB Substrate Reagent Set (BD OptEIA™, BD Biosciences Pharmingen, San Jose, CA, United States) and using human MPO (human polymorphonuclear leukocyte, EMD Millipore Corp., Billerica, MA, United States) as a standard.

### 2.7 Network pharmacology analysis

#### 2.7.1 Screening of potential bioactive components of BZYQT

The potential bioactive components of BZYQT were screened out according to our previous studies on the chemical and metabolic profiles of BZYQT (Hu et al., 2019; Liu et al., 2019). Compounds detected in bio-samples after oral administration of BZYQT and showed a relatively higher exposure were considered to be potential bioactive components (defined as BZYQT-related components).

#### 2.7.2 Target prediction of BZYQT-related components, pulmonary inflammation, and immunomodulatory effect

A systematic approach based on information integration and text-mining was applied to discover the targets of BZYQT. The most likely biological targets were retrieved from the Traditional Chinese Medicine Systems Pharmacology database (TCMSP, https://tcmspw.com/tcmsp.php), Swiss Target Prediction (http://www.swisstargetprediction.ch/), and Similarity ensemble approach (SEA, http://sea.bkslab.org/) (Pang et al., 2018; Liu et al., 2020).

The targets related to pulmonary inflammation and immunomodulatory effect were obtained from GeneCards (https://www.genecards.org/), DisGeNET (https://www.disgenet.org/), and Online Mendelian Inheritance in Man database (OMIM, https://omim.org/) (Piñero et al., 2015; Liu et al., 2020).

Finally, the common targets shared by BZYQT-related components, pulmonary inflammation, and immunomodulatory effect were screened out as the targets of BZYQT for the treatment of pulmonary inflammation.

#### 2.7.3 Network construction and pathway analysis

Cytoscape 3.8.0 software was used to create the compound-target-pathway network. On the Metascape platform, a KEGG pathway enrichment analysis of BZYQT-related targets for the treatment of pulmonary inflammation was performed (https://metascape.org/). The species was set to “*H. sapiens,*” and the parameter of Pathway & Process Enrichment were set as follows: Min Overlap: 3, *p-Value* Cutoff: 0.01, Min Enrichment: 1.5.

### 2.8 Statistical analysis

All data were expressed as mean ± SD. One-way ANOVA followed by Dunnett’s multiple comparisons test was performed for statistical analysis. *p* < 0.05 was regarded significant difference, while *p* < 0.1 was defined as tending to be significant.

## 3 Results

### 3.1 BZYQT attenuates poly (I:C)-induced neutrophils infiltration and macrophages recruitment in the lung of mice

**FIGURE 2:**
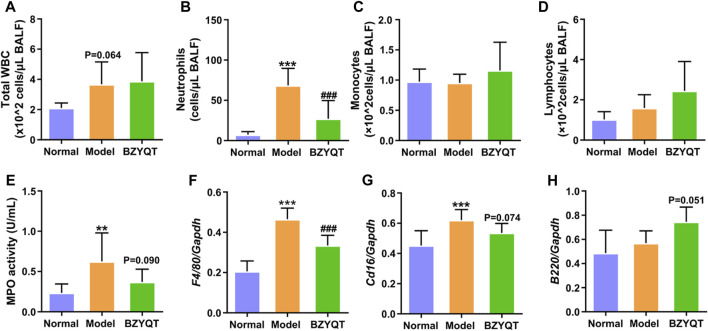
Immunomodulatory effects of BZYQT on the pulmonary immune cell infiltration induced by poly (I:C). BZYQT was protectively administered for 3 days (*n* = 7 or 8). The number of **(A)** total WBCs, **(B)** neutrophils, **(C)** lymphocytes, and **(D)** monocytes in the BALF; **(E)** MPO activity in the BALF; the mRNA expression of **(F)** *Ptprc (B220)*, **(G)** *Adgre1* (*F4/80*), and **(H)** *Cd16* in the lung. Data were shown as mean ± SD, and analyzed by one-way ANOVA; differences identified using Dunnett’s multiple comparisons test: **p < 0.01, ***p < 0.001 compared with normal group; ^###^ *p* < 0.001 compared with model group.

In order to explore the immunomodulatory effect of BZYQT on pulmonary inflammation, mice were protectively administrated with BZYQT half an hour before poly (I:C) intranasal inoculation. As Figures 2A,B,E exhibited, increases in total WBCs, neutrophils, and MPO activity were induced by poly (I:C) inoculation, suggesting successfully established pulmonary inflammation model, while protective oral administration of BZYQT for 3 days significantly suppressed the neutrophil infiltration (*p* < 0.001). With the reduction of neutrophils, MPO activity in the BALF had a tendency to decrease (*p* = 0.090). As macrophages play a major role in the development of inflammation, the gene expression of *Adgre1* (encoding the F4/80, a macrophage marker) in the lung (Waddell et al., 2018) was measured by qPCR. Although no significant influx of monocytes was observed in the BALF, the qPCR results of *F4/80* indicated that poly (I:C) triggered a large number of macrophages to accumulate in the lung, and BZYQT prevented this process (*p* < 0.001) (Figure 2F). Since macrophages can polarize into two subsets (M1 and M2) in response to inflammation, we next measured the gene expression of M1 macrophage marker (*Cd16*) and M2 macrophage marker (*Arg1*). And the results indicated that a decreased trend of *Cd16* expression was found after BZYQT treatment (*p* = 0.074, Figure 2G), while the *Arg1* expression remained unchanged (Supplementary Figure S1). Furthermore, oral administration of BZYQT for 3 days presented a tendency to up-regulate the mRNA expression of B lymphocytes marker, *B220* (encoded by the *Ptprc* locus) (*p* = 0.051, compared with the model group) (Figure 2H).

### 3.2 BZYQT ameliorates poly (I:C)-induced pulmonary inflammation

**FIGURE 3:**
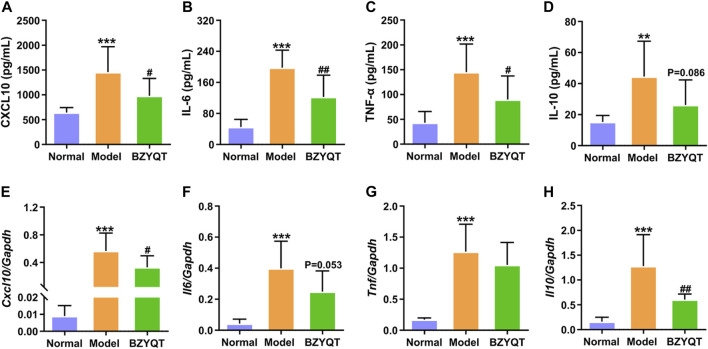
Anti-inflammatory effects of BZYQT on the pulmonary inflammation induced by poly (I:C). BZYQT was protectively administered for 3 days (*n* = 7 or 8). Contents of **(A)** CXCL10, **(B)** IL-6, **(C)** TNF-α, and **(D)** IL-10 in the BALF; mRNA expression of **(E)** *Cxcl10*, **(F)** *Il6*, **(G)** *Tnf*, and **(H)** *Il10* in the lung. Data were shown as mean ± SD, and analyzed by one-way ANOVA; differences identified using Dunnett’s multiple comparisons test: **p < 0.01, ***p < 0.001 compared with normal group; ^#^ *p* < 0.05, ^##^ *p* < 0.01, ^###^ *p* < 0.001 compared with model group.

We further evaluated the cytokines and chemokines levels in the BALF and lung tissue. As presented in Figure 3, poly (I:C) intranasal inoculation elicited elevated protein and gene expressions of CXCL10, IL-6, TNF-α, whereas the protein levels of these inflammatory mediators were significantly reduced by treatment with BZYQT (*p* < 0.05 or <0.01). The gene level of *Cxcl10* was also dramatically decreased by BZYQT, and it gave a tendency to decrease the *Il6* gene expression (*p* = 0.053). As the inflammatory response was gradually suppressed by BZYQT, the gene expression of IL-10 markedly reduced (*p* < 0.01) and its protein content tended to decrease (*p* = 0.086). These results upheld the anti-inflammatory activity of BZYQT in pulmonary inflammation.

### 3.3 BZYQT reduces the levels of inflammatory chemokines and cytokines in poly (I:C)-challenged BEAS-2B cells

**FIGURE 4:**
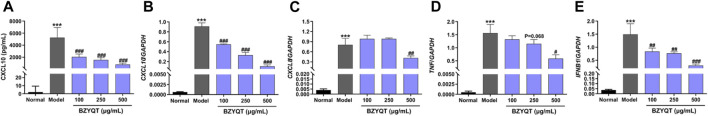
Effect of BZYQT on the protein content and mRNA expressions of inflammatory chemokines and cytokines challenged by poly (I:C) in BEAS-2B cells. The protein content of **(A)** CXCL10 in the cell supernatants; mRNA expression of **(B)** *CXCL10*, **(C)** *CXCL8*, **(D)** *TNF*, and **(E)** *IFNB1* in the lung. Data were shown as mean ± SD (*n* = 3) and analyzed by one-way ANOVA; differences identified using Dunnett’s multiple comparisons test: ***p < 0.001 compared with normal group; ^#^ *p* < 0.05, ^##^ *p* < 0.01, ^###^ *p* < 0.001 compared with model group.

In an effort to further elucidate the effects of BZYQT, inflammatory response triggered by poly (I:C) to the normal human bronchial/pulmonary epithelial cell line, BEAS-2B, was chosen for investigation, because airway epithelia are an important source of inflammatory cytokines and chemokines, and directly interact with inoculated poly (I:C). Firstly, a MTT assay was used to evaluate the cytotoxicity of BZYQT, and the results indicated that there was no significant cytotoxicity after cells treated with BZYQT at concentrations ranging from 1 to 1000 μg/ml (Supplementary Figure S2). Similarly, the increase in CXCL10 triggered by poly (I:C) was tremendously inhibited in a dose-dependent manner following BZYQT treatment at concentrations of 100, 250, and 500 μg/ml (*p* < 0.001, Figures 4A,B). However, only 500 μg/ml of BZYQT was in a position to significantly reduce the *CXCL8* and *TNF* expression (Figures 4C,D). Furthermore, BZYQT has a dose-dependent inhibitory effect on the *IFNB1* expression stimulated by poly (I:C) (Figure 4E), while there were no changes in the expression of *CXCL1*, *CXCL2*, *IL6*, *IL1B* after BZYQT treatment (data not shown).

### 3.4 Network pharmacological analysis of BZYQT on pulmonary inflammation

#### 3.4.1 Potential bioactive components in BZYQT

According to the theory of serum pharmacochemistry, bioavailable components absorbed in the bloodstream can be considered potential bioactive components (He et al., 2018). Additionally, evidence also suggests that poor oral bioavailable components may play therapeutic benefits by influencing the intestinal system (Xu et al., 2021). Therefore, based on our previous research about the chemical and *in vivo* metabolic profiles of BZYQT, and its xenobiotics analysis in the small intestinal contents (Supplemetary Table S3, Supplementary Figure S3), 46 compounds including flavonoids, triterpenoids, lactone, alkaloid, *etc.*, that derived from a set of 10 composed medicinal herbs were screened out as the potential bioactive components of BZYQT (Hu et al., 2019; Liu et al., 2019) (Table 1). It should be noted that although lobetyolin was not detected in bio-samples following oral administration of BZYQT under current conditions, it was also considered as a potential bioactive component due to its immunomodulatory activity (Gao et al., 2019), and senkyunolide H was screened out because its metabolites could be detected in the urine (Hu et al., 2019).

**TABLE 1:** Detailed information of 46 potential bioactive components of BZYQT.

No.	Compounds	Bio-samples	Structure types	Herbal medicines
A1	3β-Hydroxy atractylone	U	Lactone	AMR, BR
A2	Atractylenolide I	P, U, I	Lactone	AMR, BR
A3	Atractylenolide II	P, U	Lactone	AMR, BR
A4	Atractylenolide III	P, U	Lactone	AMR, BR
B1	Calycosin	P, U, I	Flavonoid	AR, GR
B2	Formononetin	P, U, I	Flavonoid	AR, GR
B3	Ononin	I	Flavonoid	AR, GR
B4	Liquiritigenin	P, U, I	Flavonoid	GR, AR
B5	Isoliquiritigenin	P, U, I	Flavonoid	GR, AR
C1	Ferulic acid	U	Organic acid	SM, ASR
BR1	Saikosaponin A	U, I	Triterpenoids	BR
BR2	Saikosaponin D	I	Triterpenoids	BR
BR3	2″-*O*-Acetylsaikosaponin A	I	Triterpenoids	BR
BR4	2″-*O*-Acetylsaikosaponin D	I	Triterpenoids	BR
BR5	2″-*O*-Acetyl-saikosaponin B2	I	Triterpenoids	BR
CRP1	Nobiletin	P, U, I	Flavonoid	CRP
CRP2	3,5,6,7,8,3′,4′-Heptamethoxyflavone	P, U, I	Flavonoid	CRP
CRP3	Hesperidin	I	Flavonoid	CRP
CRP4	Narirutin	P, I	Flavonoid	CRP
CRP5	Citrusin III	I	Alkaloid	CRP
CRP6	Natsudaidain-3-O-[3-hydroxy-3-methylglutarate (1→6)]-glucoside	I	Flavonoid	CRP
CRP7	Tangeretin	P, U, I	Flavonoid	CRP
ASR1	Z-ligustilide	P, U	Lactone	ASR
ASR2	Senkyunolide H	n.d	Lactone	ASR
ASR3	Butylphthalide	P, U	Lactone	ASR
CR1	Codonopsine	I	Alkaloid	CR
CR2	Lobetyolin	n.d	Other	CR
CR3	Codonopsinol	I	Alkaloid	CR
JF1	Magnoflorine	I	Alkaloid	JF
GR1	Licoricesaponin H2	I	Triterpenoids	GR
GR2	Liquiritin	I	Flavonoid	GR
GR3	Licoricesaponin G2	U, I	Triterpenoids	GR
GR4	Licoricesaponin A3	I	Triterpenoids	GR
GR5	18*β*-Glycyrrhizic acid	I	Triterpenoids	GR
GR6	Glycyrrhetinic acid	P, U, I	Triterpenoids	GR
GR7	Licoricesaponin K2	I	Triterpenoids	GR
AR1	Calycosin-7-*O*-*β*-d-glucoside	I	Flavonoid	AR
AR2	Astragaloside IV	I	Triterpenoids	AR
AR3	Astragaloside VII	I	Triterpenoids	AR
AR4	Astragaloside III	I	Triterpenoids	AR
AR5	Astragaloside II	I	Triterpenoids	AR
ZRR1	6-Gingerol	U	Gingerol	ZRR
CiR1	Cimigenoside	P, U, I	Triterpenoids	CiR
CiR2	7,8-Didehydrocimigenol-3-*O*-*β*-d-xylopyranoside	P, U, I	Triterpenoids	CiR
CiR3	24-*epi*-7,8-Didehydrocimigenol-3-*O*-*β*-d-xylopyranoside	P, U, I	Triterpenoids	CiR
CiR4	7,8-Didehydro-25-anhydrocimigenol-3-*O*-*β*-d-xylopyranoside	P, U	Triterpenoids	CiR

Notes: P, U, and I indicate plasma, urine, and small intestinal contents after oral administration of BZYQT, respectively. n.d. indicates that it could not be detected in bio-samples but was presented in BZYQT water extract; AR, *astragali radix* praeparata cum melle; AMR, *atractylodis macrocephalae rhizoma*; CR, *codonopsis radix*; GR, *glycyrrhizae radix et rhizoma* praeparata cum; CRP, *Citri Reticulatae Pericarpium*; ASR, *angelicae sinensis radix*; CiR, *cimicifugae rhizoma*, BR, *bupleuri radix*; JF, *jujubae fructus*; ZRR, *zingiberis rhizoma recens.*

#### 3.4.2 Putative immune-related targets of BZYQT for pulmonary inflammation

**FIGURE 5:**
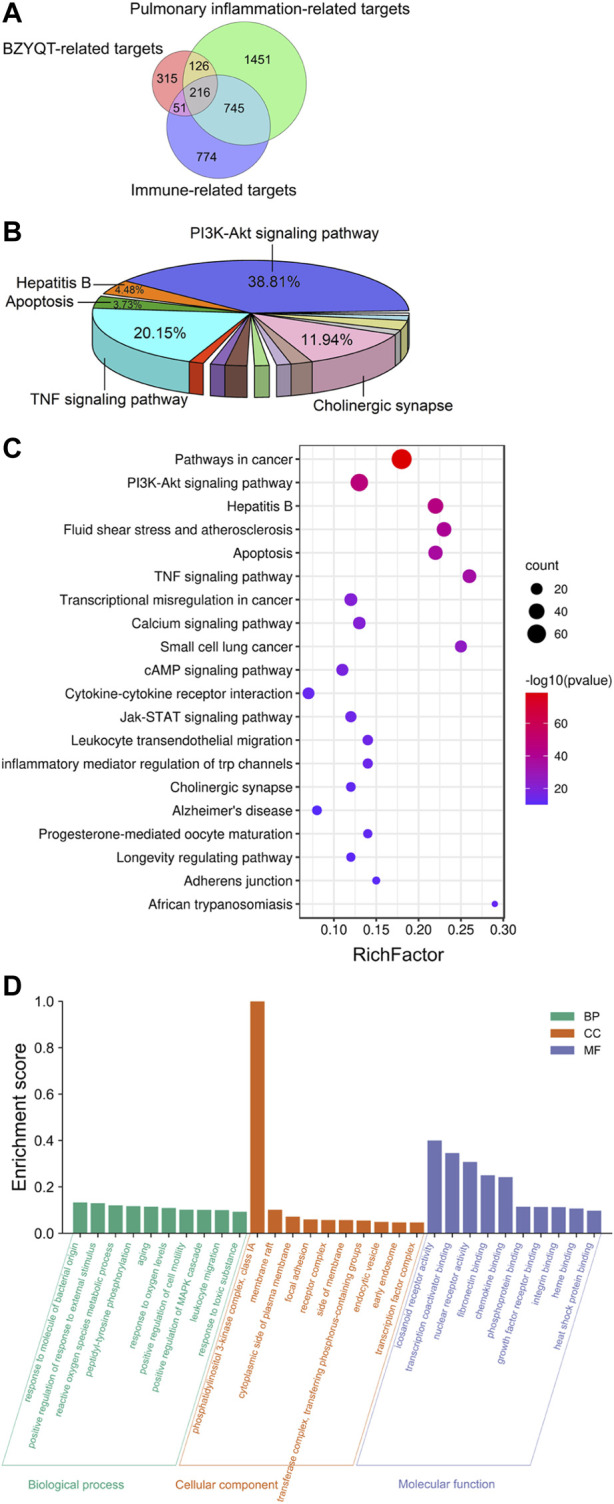
Network pharmacology analysis of the immunomodulatory effects of BZYQT for pulmonary inflammation. **(A)** Venn diagram of BZYQT-related targets, lung inflammation-related targets, and immune-related targets; **(B)** Pie chart of KEGG pathway cluster of BZYQT after hierarchical clustering analysis; **(C)** Bubble chart of 20 KEGG pathway cluster of BZYQT (the size of the circle means the gene number that hit in the pathway; the rich factor is the ratio between the observed counts and the counts expected by chance); **(D)** Top 10 terms of GO biological process (BP), cellular component (CC), and molecular function (MF) enrichment analysis.

After transforming protein targets into genes by using the Uniprot database, and removing the non-human genes and duplicates, 708 targets of BZYQT-related components were obtained from the TCMSP, Swiss Target Prediction, and SEA database; 2,538 and 1786 gene targets associated with pulmonary inflammation and immunomodulation were collected by searching the GeneCards, DisGeNET, and OMIM databases. Finally, as shown in Figure 5A, 216 targets of BZYQT against pulmonary inflammation were obtained by combining these three parts of targets of BZYQT-related components, pulmonary inflammation, and immunomodulation.

#### 3.4.3 KEGG and GO enrichment analysis of overlapping targets

To explore the biological functions of BZYQT, KEGG pathway and GO enrichment analyses of 216 overlapping targets were performed by using Metascape, an online tool. Terms with a *p* < 0.01, a minimum count of three, and an enrichment factor >1.5 (the enrichment factor is the ratio between the observed counts and the counts expected by chance) were clustered. As a result, 134 pathways were gathered and hierarchically clustered into a tree that included 20 pathways based on Kappa-statistical similarities among their gene membership. As exhibited in Figure 5B, approximately 38.81% of the 134 pathways were related to the PI3K-Akt signaling pathway, while 20.15% were involved in TNF signaling pathway, followed by cholinergic synapse, hepatitis B, and apoptosis. Enriched top 20 pathway clusters were displayed in the bubble chart (Figure 5C). Through consulting literature and combining experimental results, the PI3K-Akt signaling pathway, the TNF signaling pathway, leukocyte transendothelial migration, and cytokine-cytokine receptor interaction were supposed to be involved in the immunomodulatory effects of BZYQT on poly (I:C)-induced pulmonary inflammation.

Figure 5D depicted the top 10 GO enrichment terms for each category. Targets of BZYQT were discovered to be markedly involved in the immune process and inflammatory response, such as the biological process of response to oxygen levels, positive regulation of cell motility, and leukocyte migration. In addition, membrane raft, focal adhesion, and receptor complex were also proposed being associated with BZYQT’s effect. Further, the molecular function of BZYQT was suggested to be related to fibronectin binding, chemokine binding and integrin binding, etc.

#### 3.4.4 Construction of the compound-target-pathway network

**FIGURE 6:**
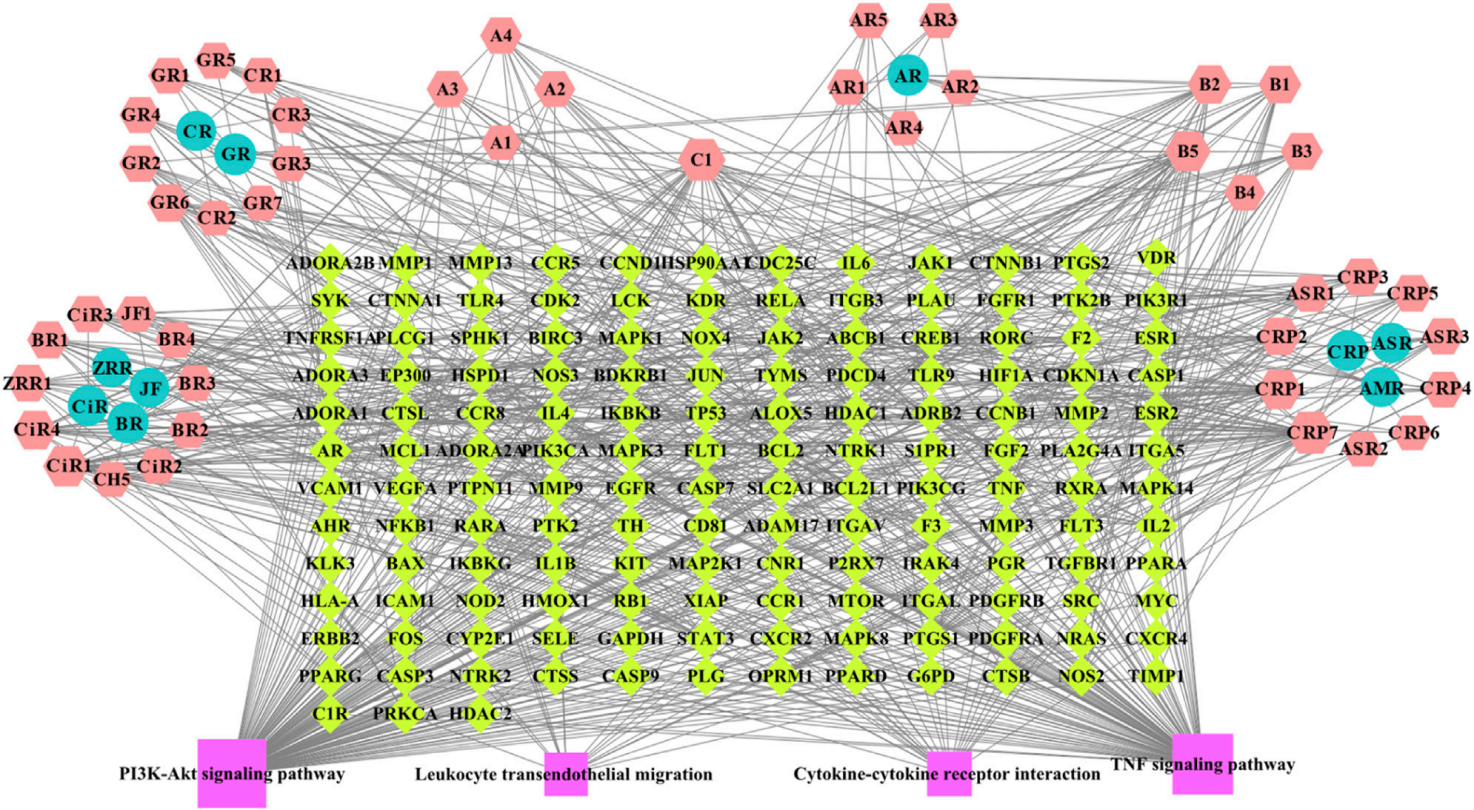
BZYQT-related “compound-target-pathway” network (hexagons mean potential bioactive components of BZYQT; circles mean composed herbal medicines of BZYQT; rhombuses mean immune-related targets and pulmonary inflammation-related targets; squares mean core signaling pathway).

A network including 46 BZYQT-related potential bioactive components, 10 composed herbal medicines of BZYQT, 146 targets, and four core signaling pathways, were constructed by using Cytoscape 3.8.0 (Figure 6). Among the 46 potential bioactive components, nine compounds had degree values greater than or equal to 15, including tangeretin (CPR7), ferulic acid (C1), cimigenoside (CiR1), isoliquiritigenin (B5), nobiletin (CPR1), 7,8-didehydro-25-anhydrocimigenol-3-*O*-*β*-d-xylopyranoside (CiR4), formononetin (B2), calycosin (B1), glycyrrhetinic acid (GR6). Furthermore, multiple targets interacted with BZYQT-related components.

### 3.5 BZYQT shows an immunomodulatory effect on the pulmonary leukocyte transendothelial migration induced by poly (I:C)

**FIGURE 7:**
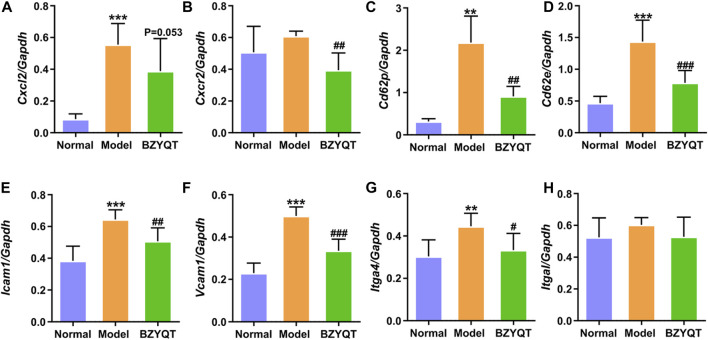
Effect of BZYQT on the pulmonary leukocyte transendothelial migration induced by poly (I:C). The mRNA expression of **(A)** *Cxcl2*, **(B)** *Cxcr2*, **(C)** *Cd62p*, **(D)** *Cd62e*, **(E)** *Icam1*, **(F)** *Vcam1*, and **(G)** *Itga4*, and **(H)** *Itgal* in the lung (*n* = 7 or 8). Data were shown as mean ± SD (*n* = 8), and analyzed by one-way ANOVA; differences identified using Dunnett’s multiple comparisons test: **p < 0.01, ***p < 0.001 compared with normal group; ^#^ *p* < 0.05, ^##^ *p* < 0.01, ^###^ *p* < 0.001 compared with model group.

From the above results of *in vivo* and *in vitro* experiments, KEGG and GO analysis in network pharmacology, the immune mechanism of BZYQT was suggested to be related to the interference of leukocyte transendothelial migration. To verify this result, gene expression of molecules that regulate paracellular transmigration, such as cell-adhesion molecules and related chemokines in the lung tissue of poly (I:C)-induced mouse model were measured. As exhibited in Figures 7A,B, BZYQT presented a tendency to down-regulate the expression of neutrophil-related chemokine, *Cxcl2*, which was triggered by poly (I:C) inoculation, and its receptor, *Cxcr2* was significantly down-regulated. In addition, the levels of selectins (e.g., E- and P-selectin) and integrins (e.g., VLA-4 and LFA-1), two major adhesion receptor families that mediate the leukocyte-adhesion cascade, as well as associated endothelial cell ligands (e.g., VCAM-1 and ICAM-1), were also analyzed. Undoubtedly, the up-regulated *Cd62e* (E-selectin), *Cd62p* (P-selectin), *Icam1*, *Vcam1*, and *Itga4* (α_4_ subunit of VLA-4) were dramatically down-regulated when mice treated with BZYQT at a dose of 1.5 g/kg/day for 3 days (Figures 7C–G), but there is no significant difference in the expression of *Itgal* (α_L_ subunit of LFA-1, Figure 7H). As we known, leukocyte migration from circulating blood to the sites of infection and injury is a fundamental immune response with the principal aim of eliminating the primary inflammatory trigger and repairing the injured tissues (Nourshargh and Alon, 2014), therefore, these results suggested that BZYQT could reduce the pulmonary inflammation by regulating leukocyte transendothelial migration.

Given the above findings, we sought to screen the bioactive components of BZYQT against pulmonary inflammation by integrating network pharmacology and experimental results. According to the relationship between the “compounds” and “targets,” and literature, isoliquiritigenin, Z-ligustilide, atractylenolide I, atractylenolide III, formononetin, ferulic acid, hesperidin, and cimigenoside were screened out as the bioactive components of BZYQT because their predicted targets such as *IL6, TNF, ICAM1, SELE* (CD62E in mouse), *VCAM1, CXCR2* were related to the genes that BZYQT regulated (Li et al., 2007; Zhang et al., 2015; Zhang et al., 2018; Xie et al., 2020; Yi et al., 2020; Hu, et al., 2021; Li et al., 2021; de Souza et al., 2022).

## 4 Discussion

Numerous studies have shown that BZYQT possesses a variety of immunomodulatory effects, such as potentiation of the NK cell activity in elderly persons (Kuroiwa et al., 2004), reduction of IgE, Th2-related cytokines levels, and prevention of the eosinophil infiltration in asthmatic mice (Yang and Yu, 2008), suppression of the macrophages in emphysema mice (Isago et al., 2021), stimulation of the G-CSF in intestinal epithelial cells (Matsumoto et al., 2010), etc. We herein demonstrated that BZYQT exerted a potent immunomodulatory effect in poly (I:C)-induced pulmonary inflammation by integrating network pharmacology and experiments.

Poly (I:C), a synthetic analogue of double-stranded RNA, is widely used to mimic viral infections. Upon poly (I:C) recognized by Toll-like receptor 3 (TLR3), a series of pathways such as the NF-κB signaling pathway will be activated and then trigger the production of inflammatory cytokines and chemokines, such as IL-6, TNF-α, CXCL10, CXCL1, and recruit the WBCs, especially neutrophils to the inoculated tissue (Stowell et al., 2009). There are four stages including rolling adhesion, tight binding, diapedesis, and migration for neutrophils to cross the blood vessel wall and migrate to the inflamed tissue, which involves adhesive interactions that are regulated by cytokines and chemokines (Nourshargh and Alon, 2014). Selectins, such as E- and P-selectin, expressed by endothelial cells, are important adhesion molecules for neutrophils and monocytes that adhere reversibly to the vessel wall and rolling along the endothelium. Adhesion molecules appeared on endothelium, such as ICAM-1 and VCAM-1, interacting with integrins LFA-1 and VLA-1 is an essential step for neutrophils attaching firmly to endothelium under the situation of chemokine binds to its receptors on neutrophils. After neutrophils extravasate from the endothelial wall, they migrated to the infected tissues under the guidance of chemokines.

Our experimental results showed that protective oral administration of BZYQT for 3 days significantly decreased the number of neutrophils and the gene expression of macrophages cell marker (F4/80) in the lung. With the decrease of neutrophils, the expression of CXCR2, a receptor of neutrophil chemokines (e.g., CXCL1 and CXCL2), and the production of MPO, a key granule protein expressed mainly by neutrophils, were correspondingly reduced. Both neutrophils and macrophages are recognized as inflammatory cells. It has been identified that neutrophil accumulation in the lung was a major cause of acute lung injury in many respiratory diseases (e.g., COPD, acute respiratory distress syndrome, and cystic fibrosis) as they release various inflammatory mediators (e.g., MPO) to help kill the invader, which may also damage the normal tissues (Abraham et al., 2000). Macrophages are also the source of a variety of pro-inflammatory cytokines, such as IL-6, TNF-α, and IL-1β, and they can trigger tissue damage because of the toxic activity of oxygen and nitrogen species. In inflammatory responses, it can polarize into two subsets M1 and M2. M1 macrophages are known as inflammatory macrophages that defend against pathogens and protect against dangerous stimuli, which cause chronic inflammation and tissue damage, whereas M2 macrophages are known as healing macrophages or deactivated macrophages because they affect branched morphology and angiogenesis (Italiani and Boraschi, 2014). We have revealed that the mRNA expression of M1 cell marker (*Cd16*) tended to degrade after BZYQT treatment (*p* = 0.074). However, since no specific marker has been found for accurately distinguishing M1 and M2 types, it is still difficult to make a conclusion of which type of macrophages was reduced by BZYQT, and more studies need to be further investigated. Subsequently, it was found that the ameliorated pulmonary inflammatory by BZYQT was associated with the decreased expression of CXCL10, IL-6, TNF-α, and IFN-β induced by poly (I:C). Research has proved that the increased level of CXCL10 in the lung with acute respiratory distress syndrome was largely attributed to infiltrated neutrophils, and that neutralization of CXCL10 could ameliorate lung injury induced by lipopolysaccharide (Ichikawa et al., 2013; Lang et al., 2017). Take into account these findings, BZYQT was thought to exert immunomodulatory effects on neutrophil and macrophage infiltration to attenuate lung injury.

Network pharmacology is a promising auxiliary approach to understanding the molecular mechanism and the effective components of TCM (Tao et al., 2013; Pang et al., 2018; Zhou et al., 2022). The network pharmacology analysis based on 46 BZYQT-related potential bioactive components showed that the effects of BZYQT on pulmonary inflammation were associated with the immune process and inflammatory response, and four signaling pathways including PI3K-Akt signaling pathway, TNF signaling pathway, leukocyte transendothelial migration, and cytokine-cytokine receptor interaction were screened out. Crucial targets that took part in these pathways were validated through experiments (Supplemetary Table S4), including *IL6, TNF, ICAM1, SELE* (CD62E in mouse), *VCAM1*, and *CXCR2*. Since BZYQT performed dramatical regulation of the migration process of inflammatory cells such as neutrophils and macrophages by down-regulating the levels of E-selectin, P-selectin, ICAM-1, VCAM-1, and VLA-4, and relevant cytokines and chemokines, we believe that its immunomodulatory effects on pulmonary inflammation may be related to the interference with leukocyte transendothelial migration.

In our study, 129 targets out of 216 targets were enriched in PI3K-Akt signaling pathway, which is crucial for cell growth, migration, proliferation, and metabolism, and it also contributes to the functions of immune cells such as neutrophils, lymphocytes, macrophages, etc. (Pompura and Dominguez-Villar, 2018). PI3Kγ and PI3Kδ are PI3K subunits that mainly contribute to inflammatory cell recruitment and subsequent activation (Hirsch et al., 2000; Stark et al., 2018). It was pointed out that TG100-115, a dual PI3Kγ/δ inhibitor has extended benefits for asthma and COPD (Doukas et al., 2009). Therefore, in addition to leukocyte transendothelial migration, the PI3K-Akt signaling pathway may be involved in the immunomodulatory activity of BZYQT on poly (I:C)-induced pulmonary inflammation.

Through integrating the network pharmacology and experimental results, eight compounds were screened out as the bioactive components of BZYQT against pulmonary inflammation based on the relationship of “compounds” and “targets,” and literature. Among them, cimigenoside was previously confirmed to have an obvious immunomodulatory effect on poly (I:C)-induced pulmonary inflammation (Hu et al., 2021), and the other compounds were also demonstrated with significant anti-inflammatory and immunomodulatory activities on lung inflammation (Li et al., 2007; Zhang et al., 2015; Zhang et al., 2018; Xie et al., 2020; Yi et al., 2020; Li et al., 2021; de Souza et al., 2022).

**FIGURE 8:**
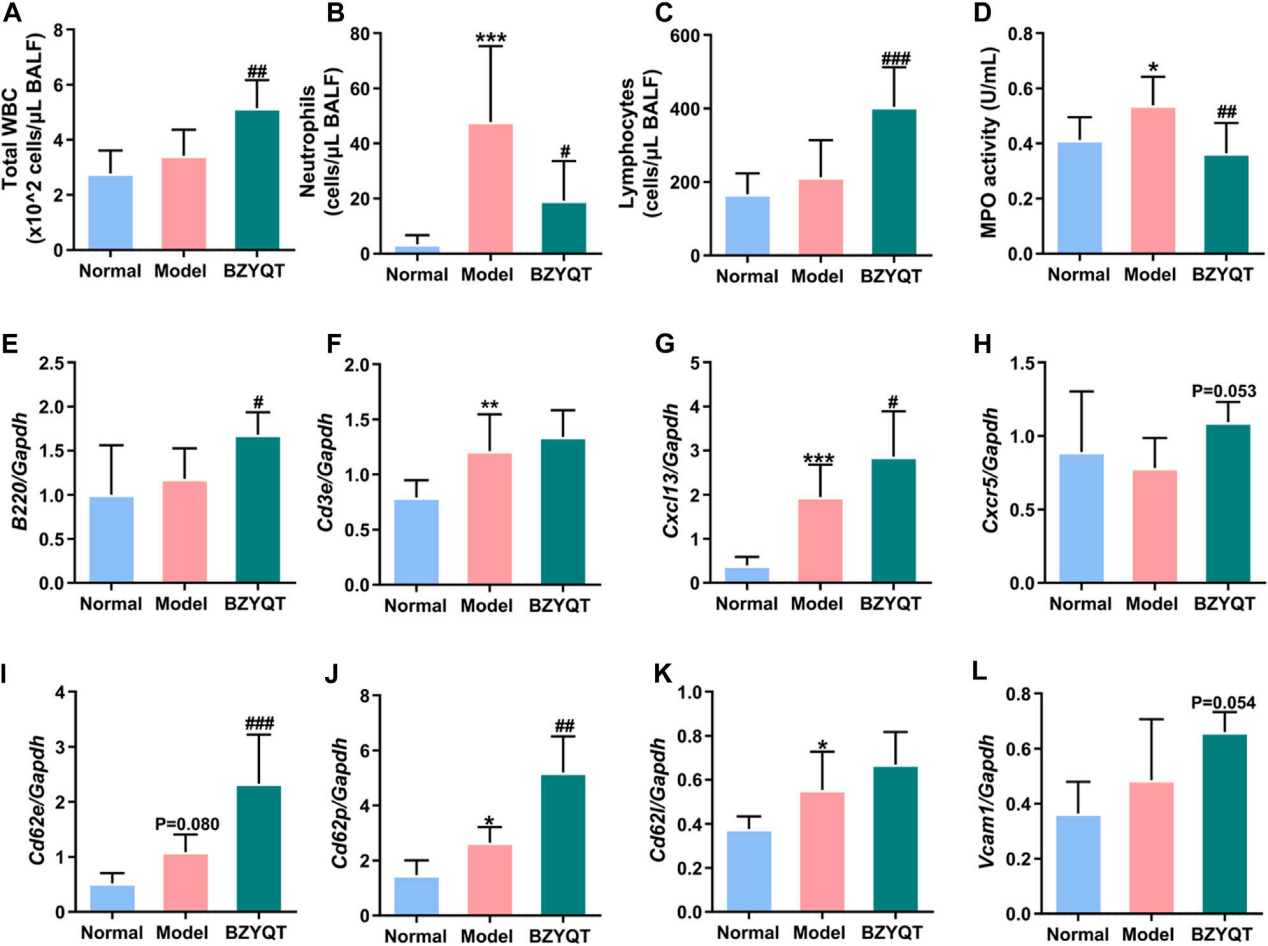
Immunomodulatory effect of BZYQT on poly (I:C)-induced pulmonary inflammation through prophylactic administration. The number of **(A)** total WBCs, **(B)** neutrophils, and **(C)** lymphocytes, **(D)** MPO activity in the BALF; the mRNA expression of **(E)** *Ptprc* (*B220*), **(F)** *Cd3e*, **(G)** *Cxcl13*, **(H)** *Cxcr5*, **(I)** *Cd62e*, **(J)** *Cd62p*, **(K)** *Cd62l*, **(L)** *Vcam1* in the lung tissue (*n* = 8 or 9). Data were shown as mean ± SD, and analyzed by one-way ANOVA; differences identified using Dunnett’s multiple comparisons test: *p < 0.05, **p < 0.01, ***p < 0.001 compared with normal group; ^#^ *p* < 0.05, ^##^ *p* < 0.01, ^###^ *p* < 0.001 compared with model group.

It is startling to discover that BZYQT showed a tendency to elevate the expression of B220, indicating that BZYQT may promote the recruitment of B lymphocytes to enhance the immune response. To further explore its effects on lymphocytes, prophylactic administration of BZYQT for 7 days on a poly (I:C)-induced mice model was investigated. As presented in Figures 8B,D, neutrophil infiltration and MPO activity were significantly reduced, while the numbers of total WBCs were dramatically increased in BZYQT group (Figure 8A). The increase in WBCs was primarily attributed to lymphocytes (Figure 8C). According to the significantly up-regulated expression of *Ptprc* (B220, B cell marker) and unchanged expression of T cell marker (*Cd3e*) in BZYQT group (Figures 8E,F), the increase of lymphocytes was proposed to be B lymphocytes rather than T lymphocytes. Accordingly, the gene expressions of molecules related to the leukocyte transendothelial migration such as a B lymphocyte chemokine, *Cxcl13*, and relevant cell-adhesion molecules, *Cd62p* and *Cd62e,* were also significantly up-regulated by BZYQT (Figures 8G,I,J). Furthermore, increased tendencies in *Cxcr5* (a receptor of CXCL13) and *Vcam1* with a *p-*value of 0.053 and 0.054 respectively were observed (Figures 8H,I). The increased B lymphocytes were speculated to be linked with the formation of iBALT (inducible bronchus-associated lymphoid tissue), which can emerge in response to infection, chronic inflammation, or autoimmunity to provide a niche for B-cell education and T-cell priming to boost protective immunity against respiratory pathogens (Foo and Phipps, 2010).

**FIGURE 9:**
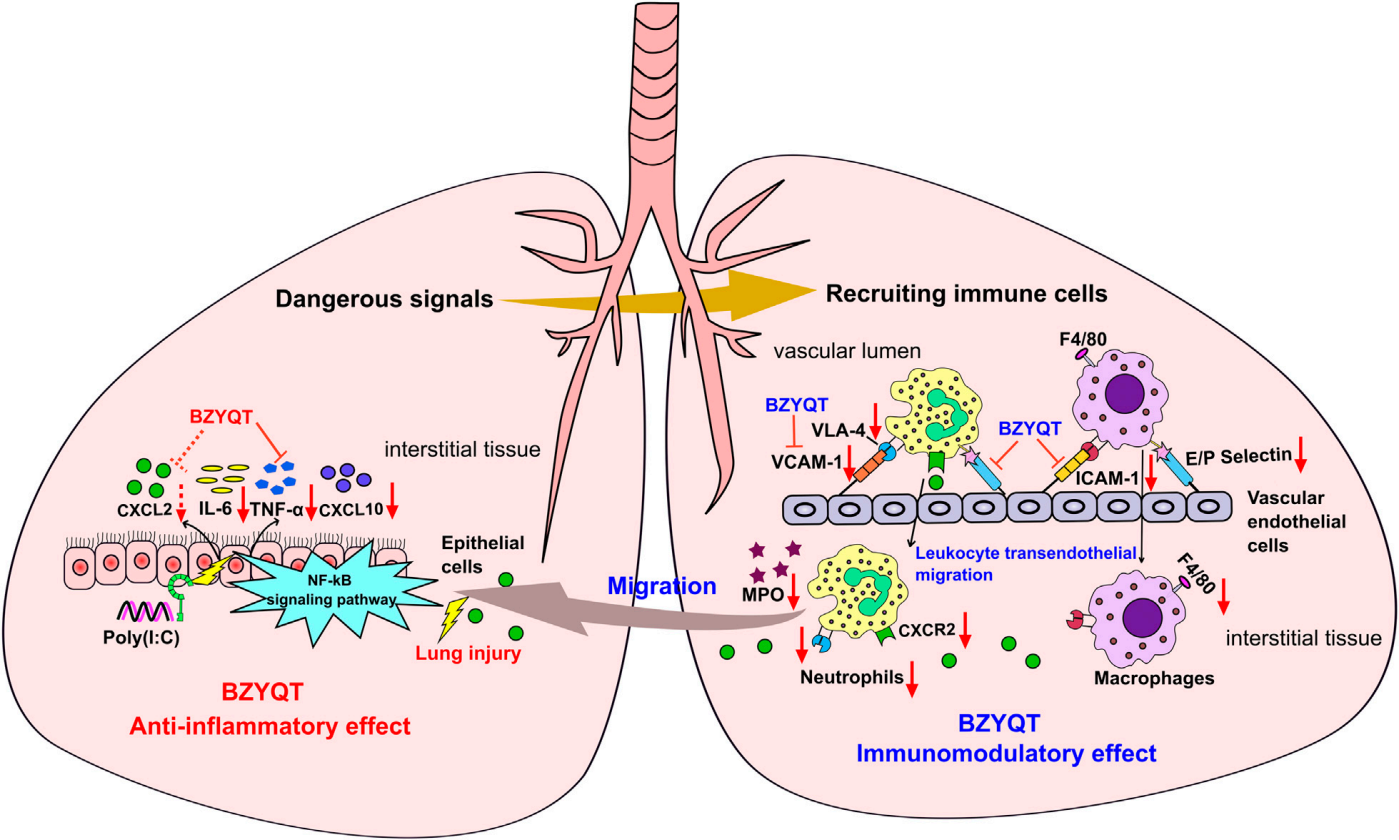
Potential mechanism of BZYQT for the alleviation of pulmonary inflammation caused by poly (I:C). The dashed line (arrow) indicates a trend.

In view of the above-mentioned facts, the immunomodulatory effect of BZYQT for the treatment of pulmonary inflammation was summarized. As shown in Figure 9, poly (I:C) inoculation triggers the release of abundant proinflammatory mediators, which as dangerous signals to stimulate leukocytes (e.g., neutrophils and macrophages) and vascular endothelial cells, and initiate a cascade transendothelial migration response; then, immune cells were recruited into the inflamed site under the induction of chemokines. BZYQT oral administration reduced the levels of CXCL10, TNF-α, IL-6, and CXCL2, and prevented the neutrophils and macrophages infiltration through down-regulating the cell-adhesion molecules such as ICAM-1, VCAM-1, E- and P-selectin. As a result, the contents of anti-inflammatory cytokine IL-10 and neutrophil product MPO were reduced, and the lung injury was alleviated.

It has been found that the molecular responses in the airway caused by poly (I:C) were directly related to the observation in the lungs of COPD patients (Harris et al., 2013). Moreover, researchers discovered that the poly (I:C)-induced lung dysfunction was similar to the respiratory syncytial virus (RSV) in BALB/c mice (Aeffner et al., 2011). A recent report indicated that TLR3 was involved in the control of innate immunity during lung SARS-CoV-2 infection (Bortolotti et al., 2021). Therefore, our present research may aid in elucidating the probable mechanism of BZYQT in the improvement of COPD and other respiratory diseases caused by viral infections.

## 5 Conclusion

Collectively, the immunomodulatory effect BZYQT on poly (I:C)-induced pulmonary inflammation was deciphered by integrating network pharmacology analysis and experimental verification, which was demonstrated to be closely associated with anti-inflammatory activity and interference of leukocyte tranendothelia migration. BZYQT remarkably reduced the neutrophils and macrophages infiltration by regulating the proinflammatory cytokines TNF-α, IL-6, the chemokines CXCL10 and the cell-adhesion molecules E- and P-selectin, VCAM-1, and ICAM-1. Moreover, isoliquiritigenin, Z-ligustilide, atractylenolide I, atractylenolide III, formononetin, ferulic acid, hesperidin, and cimigenoside were presumed as the bioactive components of BZYQT. Our findings would provide a potential mechanism of BZYQT for relieving the exacerbation of COPD and respiratory tract viral infection, which enrich our knowledge of its efficacy on the respiratory system.

## Data Availability

The original contributions presented in the study are included in the article/Supplementary Material, further inquiries can be directed to the corresponding authors.

## Abbreviations

BALF, bronchoalveolar lavage fluid; BSA, bovine serum albumin; BZYQT, Bu-Zhong-Yi-Qi-Tang; COPD, chronic obstructive pulmonary disease; MPO, myeloperoxidase; PBS, phosphate buffered saline; Poly(I:C), polyinosinic-polycytidylic acid; TCM, traditional Chinese medicine; WBC, white blood cell.
