# Supplementary material for: Integrating network pharmacology and experimental verification to decipher the immunomodulatory effect of Bu-Zhong-Yi-Qi-Tang against poly (I:C)-induced pulmonary inflammation
Source: Front Pharmacol. 2022 Oct 11;13:1015486. doi: 10.3389/fphar.2022.1015486 (PMC9592993; doi:10.3389/fphar.2022.1015486)
Supplement: Supplementary file 1 [file DataSheet1.docx]

**Supporting Information**

**Figure Captions**

**Fig. S1.** The mRNA expression of *Arg1* in the lung of mice after protective oral administration of BZYQT.

**Fig. S2.** Cytotoxicity of BZYQT on BEAS-2B cells at concentrations of 1, 10, 100, 500, 1000 μg/mL.

**Fig. S3.** Extracted ion chromatograms (EICs) of xenobiotics in small intestinal contents after oral administration of BZYQT

**Table Captions**

**Table S1.** The sequences of murine primer used for quantitative PCR.

**Table S2.** The sequences of human primer used for quantitative PCR.

**Table S3.** UPLC-Q-TOF/MS data of prototypes in rat small intestinal contents after oral administration of BZYQT extract.

**Table S4.** Core signaling pathways might be related to the immunomodulatory effect of BZYQT for the treatment of poly(I:C)-induced lung inflammation


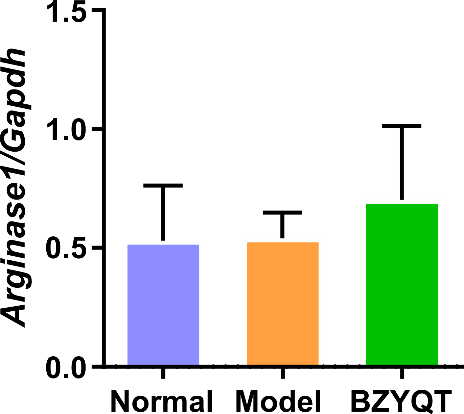


**Fig. S1. The mRNA expression of *Arg1* in the lung of mice after protective oral administration of BZYQT for 3 days.** Data were shown as mean ± SD (n=7 or 8), and analyzed by one-way ANOVA, and differences identified using Dunnett's multiple comparisons test.


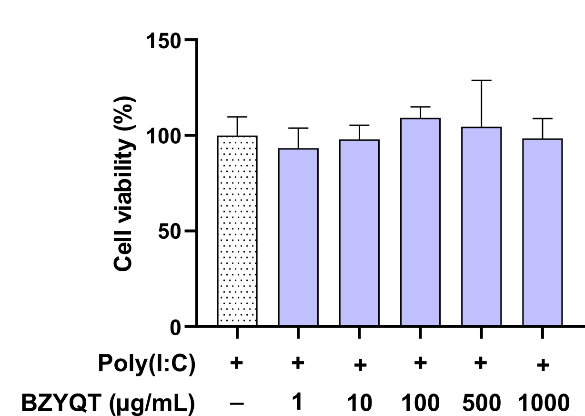


**Fig. S2.** **Cytotoxicity of BZYQT on BEAS-2B cells at concentrations of 1, 10, 100, 500, 1000 μg/mL.** Data were shown as mean ± SD (n=4).

**
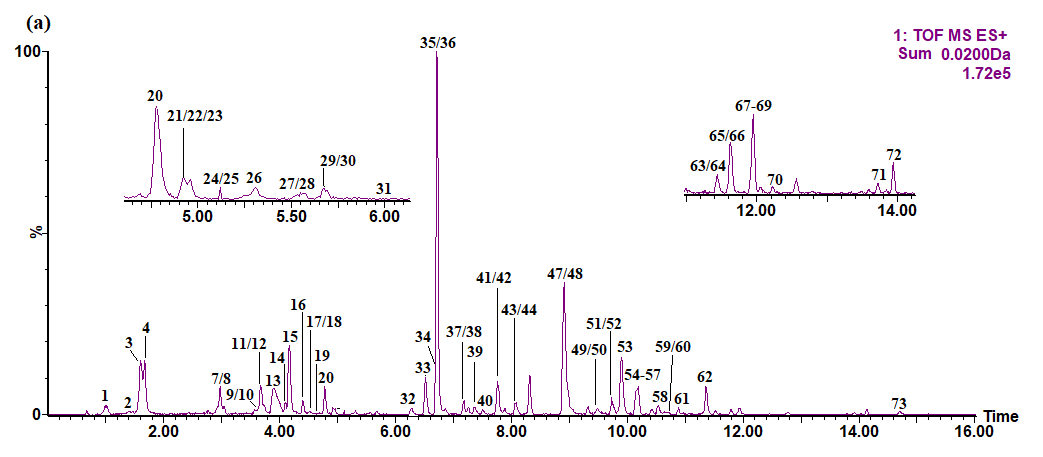

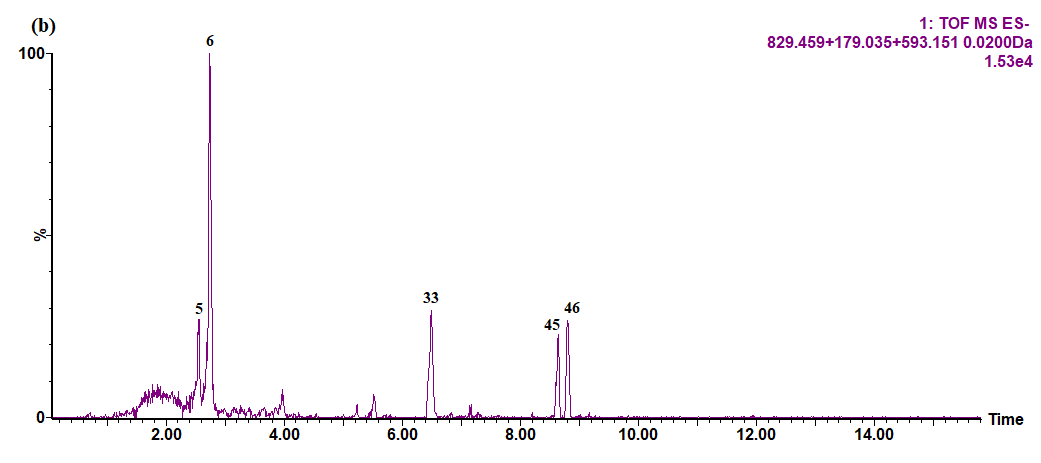
**

**Fig. S3.** **Extracted ion chromatograms (EICs) of prototypes in small intestinal contents after oral administration of BZYQT.** (a) positive ion mode; (b) negative ion mode.

**Note:** Rats were orally administrated with BZYQT at a dose of 20 g/kg/day for 3 consecutive days. Small intestinal contents were collected at 0.25, 0.5, 1, 2, 4 h after the last oral administration, and the samples were pretreated according to the pretreatment of feces that mentioned in our previous research of the metabolic profile of BZYQT (Hu et al., 2019). The prototypes in the small intestinal contents were identified based on the retention time and mass fragmentation of the compounds characterized in BZYQT extracts (Liu et al., 2019).

**Table S1.** The sequences of murine primer used for quantitative PCR

| **Oligo name** | **Forward** | **Reverse** |
| --- | --- | --- |
| *Arg1* | GTTCTGGGAGGCCTATCTTACA | TCCCAAGAGTTGGGTTCACT |
| *Ptprc* (*B220*) | TCTTTGTCACAGGGCAAACAC | AGATGCTTGGGGGTGTGGATT |
| *Cd16* | TTGCTTTTGCAGACAGGCAGAG | CTTCGCACATCAGTGTCACCAT |
| *Cd3e* | ATTCTGAGAGGATGCGGTGGAA | TAGAGGGCACGTCAACTCTACA |
| *Cd62p* | ACATGCCAGTTCATGTGCGA | TTCCTGGACACTTGATGGCT |
| *Cd62e* | TTTCGGCACAGTGTGTGAGT | CAACTACCAAGGGACGGGTG |
| *Cxcl10* | TGAGGGCCATAGGGAAGCTTGAAAT | TCCGGATTCAGACATCTCTGCTCAT |
| *Cxcl13* | GGATTCAAGTTACGCCCCCT | ACCATTTGGCACGAGGATTCA |
| *Cxcl2* | GCCCAGACAGAAGTCATAGCC | TCCTCCTTTCCAGGTCAGTT |
| *Cxcr2* | TGCTCACAAACAGCGTCGTAGA | AGAGGGCATGCCAGAGCTATAA |
| *Adgre1* (*F4/80*) | TGTGCCATCATTGCGGGATT | TCTTGATGTTGCGAGAGCTG |
| G*apdh* | TGTGTCCGTCGTGGATCTGA | TTGCTGTTGAAGTCGCAGGAG |
| *Icam1* | TGCCAGTACTGCTGGTCATT | GGGCTTGTCCCTTGAGTTTT |
| *Il6* | AGACAAAGCCAGAGTCCTTCAGAGA | GGAGAGCATTGGAAATTGGGGTAGG |
| *Il10* | TTTGAATTCCCTGGGTGAGAA | GCTCCACTGCCTTGCTCTTATT |
| *Itga4* | AAGAATCCAAACCAGACCTGCGAAC | ACGATAGAGCCATTTTCTCCAGGCT |
| *Itgal* | GAATGACGCTGGCAACAGAT | TGTAACATAGGTCCCTCCAGAC |
| *Vcam1* | TGTGAAGGGATTAACGAGGC | GTGCAGGAGATAATGACGGTGT |

**Table S2.** The sequences of human primer used for quantitative PCR

| **Oligo name** | **Forward** | **Reverse** |
| --- | --- | --- |
| *GAPDH* | GCACCGTCAAGGCTGAGAAC | TGGTGAAGACGCCAGTGGA |
| *CXCL8* | ACACTGCGCCAACACAGAAATTA | TTTGCTTGAAGTTTCACTGGCATC |
| *CXCL10* | GGCCATCAAGAATTTACTGAAAGCA | TCTGTGTGGTCCATCCTTGGAA |
| *IFNB1* | ACAGGTTACCTCCGAAACTGAAGA | TTAGCCATCAGTCACTTAAACAGCA |
| *TNF* | CTGCCTGCTGCACTTTGGAG | ACATGGGCTACAGGCTTGTCACT |

**Table S3.** UPLC-Q-TOF/MS data of prototypes in rat small intestinal contents after oral administration of BZYQT extracts

| **No.** | **t_R_** | **Selected ion** | **Formula** | **M+H (mDa, ppm)** | **MS fragmentations** | **Identification** | **MS response** |
| --- | --- | --- | --- | --- | --- | --- | --- |
| 1 | 0.99 | [M+H]^+^ | C_14_H_21_NO_5_ | 284.1497 (-0.1, -0.4) | 161.0608 | Codonopsinol | + |
| 2 | 1.41 | [M+H]^+^ | C_19_H_29_NO_9_ | 416.1915 (-0.6, -1.4) | 268.1550, 186.1235 | Codonopiloside A | + |
| 3 | 1.59 | [M+H]^+^ | C_13_H_19_NO_4_ | 254.1390 (-0.2, -0.8) | 161.0600 | Codonopsinol B | + |
| 4* | 1.60 | [M+H]^+^ | C_14_H_21_NO_4_ | 268.1547 (-0.2, -0.7) | 161.0605, 121.0674, 88.0764 | Codonopsine | ++ |
| 5 | 2.55 | [M-H]^-^ | C_9_H_8_O_4_ | 179.0351 (0.3, 1.7) | 161.0237, 135.0455 | Caffeic acid | + |
| 6 | 2.76 | [M-H]^-^ | C_27_H_30_O_15_ | 593.1506 (0, 0) | 473.1085, 353.0655 | Apigenin-6,8-di-C-glucoside | ++ |
| 7* | 2.92 | [M+H]^+^ | C_28_H_32_O_16_ | 625.1772 (0.3, 0.5) | 177.0552 | Diosmetin 6,8-di-C-glucoside | + |
| 8* | 2.94 | [M+H]^+^ | C_20_H_24_NO_4_ | 342.1705 (0, 0) | 297.1130, 282.0909, 265.0877 | Magnoflorine | ++ |
| 9 | 3.56 | [M+H]^+^ | C_27_H_30_O_16_ | 611.1616 (0.8, 1.3) | 465.1036, 303.0501 | Rutin | + |
| 10* | 3.65 | [M+H]^+^ | C_22_H_22_O_10_ | 447.1291 (-0.1, -0.2) | 285.0763, 137.0230 | Calycosin-7-*O*-*β*-d-glucoside | + |
| 11 | 3.67 | [M+H]^+^ | C_26_H_30_O_13_ | 551.1771 (0.6, 1.1) | 257.0813, 137.0246 | Neoliquiritin apioside | ++ |
| 12* | 3.71 | [M+H]^+^ | C_21_H_22_O_9_ | 419.1341 (-0.1, -0.2) | 257.0812, 137.0237 | Liquiritin | + |
| 13 | 3.84 | [M+H]^+^ | C_18_H_34_O_11_ | 425.2034 (1.1, 2.6) | 263.1496 | Hexyl 2-*O*-*β*-d-glucopyranosyl-(1-6)-β-D-glucopyranoside | + |
| 14* | 4.08 | [M+H]^+^ | C_28_H_32_O_16_ | 625.1768 (-0.4, -0.6) | 317.0657 | Narcissoside | + |
| 15* | 4.16 | [M+H]^+^ | C_27_H_32_O_14_ | 581.1868 (-0.2, -0.3) | 563.1755, 435.1295, 419.1336, 401.1232, 383.1133, 273.0756 | Narirutin | ++ |
| 16 | 4.43 | [M+H]^+^ | C_25_H_24_O_13_ | 533.1295 (-0.5, -0.9) | 285.0760 | Calycosin-7-*O*-*β*-d-glucoside-6"-*O*-malonate | + |
| 17* | 4.50 | [M+H]^+^ | C_28_H_34_O_15_ | 611.1976 (-0.6, -1.0) | 465.1392, 449.1444, 431.1335, 345.0972, 303.0864 | Hesperidin | ++ |
| 18* | 4.56 | [M+H]^+^ | C_24_H_24_O_11_ | 489.1396 (-0.1, -0.2) | 285.0764 | Calycosin-acetyl-glucoside | ++ |
| 19 | 4.60 | [M+H]^+^ | C_30_H_34_O_18_ | 683.1816 (-0.7, -1.0) | 653.1722, 611.1973, 377.0872, 347.0771, 317.0670, 303.0866 | Limocitrol 3-*O*-(3-hydroxy-3-methylglutarate)-glucoside | + |
| 20* | 4.76 | [M+H]^+^ | C_26_H_30_O_13_ | 551.1769 (0.4, 0.7) | 419.1339, 257.0810, 137.0238 | Liquiritin apioside | + |
| 21 | 4.91 | [M+H]^+^ | C_26_H_30_O_13_ | 551.1774 (0.9, 1.6) | 419.1335, 257.0813 | Isoliquiritin apioside | + |
| 22 | 4.94 | [M+H]^+^ | C_21_H_22_O_9_ | 419.1335 (-0.7, -1.7) | 257.0805 | Isoliquiritin | + |
| 23 | 4.99 | [M+H]^+^ | C_24_H_24_O_11_ | 489.1392 (-0.3, -0.6) | 285.0768 | Calycosin-acetyl-glucoside | + |
| 24 | 5.16 | [M+H]^+^ | C_22_H_22_O_9_ | 431.1338 (-0.2, -0.5) | 269.0810, 253.0490, 237.0559 | Ononin | ++ |
| 25 | 5.17 | [M+H]^+^ | C_16_H_14_O_5_ | 287.0917 (-0.2, -0.7) | 269.0810 | Licochalcone B | ++ |
| 26 | 5.29 | [M+H]^+^ | C_15_H_12_O_4_ | 257.0813 (-0.1, -0.4) | 239.0724, 207.1845, 177.5375, 147.0437, 137.0236, 121.5085 | Liquiritigenin | ++ |
| 27 | 5.57 | [M+H]^+^ | C_27_H_32_O_14_ | 581.1870 (0, 0) | 419.1339, 389.0869, 361.0930 | Natsudaidain-3-*O*-*β*-d-glucoside | ++ |
| 28* | 5.61 | [M+H]^+^ | C_16_H_12_O_5_ | 285.0757 (-0.6, -2.1) | 253.0495, 213.0547, 197.0604, 137.0238 | Calycosin | + |
| 29 | 5.73 | [M+H]^+^ | C_28_H_34_O_14_ | 595.2025 (-0.2, -0.5) | 449.1438, 287.0920 | Didymin | ++ |
| 30 | 5.76 | [M+H]^+^ | C_42_H_62_O_17_ | 839.4066 (0.1, 0.1) | 663.3743, 469.3315 | 24 -Hydroxyl glycyrrhizin or isomer | + |
| 31 | 6.02 | [M+H]^+^ | C_48_H_72_O_22_ | 1001.4592 (-0.1, -0.1) | 469.3302 | 24-Hydroxyl-licorice-saponin A3 or isomer | + |
| 32 | 6.33 | [M+H]^+^ | C_36_H_53_N_7_O_9_ | 728.3982 (-0.1, -0.1) | 700.4023, 615.3134, 419.1333 | Citrusin III | + |
| 33 | 6.58 | [M+H]^+^ | C_48_H_72_O_21_ | 985.4637 (-0.7, -0.7) | 809.4310, 647.3773, 471.3479, 453.3363 | Licoricesaponin A3 | ++ |
| 34* | 6.65 | [M+H]^+^ | C_24_H_24_O_10_ | 473.1448 (0, 0) | 269.0811 | 6'-*O*-Acetyl ononin | + |
| 35 | 6.71 | [M+H]^+^ | C_47_H_78_O_19_ | 947.5222 (0.6, 0.6) | 455.3532 | Astragaloside VII | ++ |
| 36 | 6.71 | [M+H]^+^ | C_33_H_40_O_18_ | 725.2291 (-0.2, -0.3) | 419.1343, 389.0863 | Natsudaidain-3-*O*-[3-hydroxy-3-methyl-glutarate (1→6)]-glucoside | +++ |
| 37 | 7.15 | [M+H]^+^ | C_33_H_40_O_18_ | 725.2296 (-0.5, -0.7) | 419.1349 | Natsudaidain-3-*O*-(3-hydroxy-3-methyl-glutarate)-glucoside | ++ |
| 38 | 7.21 | [M+H]^+^ | C_42_H_62_O_17_ | 839.4053 (-1.2 -1.4) | 663.3754, 487.3437, 469.3311 | 22 -Hydroxyl glycyrrhizin or isomer | + |
| 39* | 7.36 | [M+H]^+^ | C_15_H_12_O_4_ | 257.0811 (-0.3, -1.2) | 137.0239 | Isoliquiritigenin | + |
| 40 | 7.51 | [M+H]^+^ | C_20_H_20_O_7_ | 373.1282 (-0.5, -1.3) | 343.0816, 277.2157, 195.1385, 149.1326 | Isosinensetin | + |
| 41* | 7.72 | [M+H]^+^ | C_16_H_12_O_4_ | 269.0810 (-0.4, -1.5) | 253.0508, 237.0529, 213.0925, 197.0594, 137.0239 | Formononetin | ++ |
| 42 | 7.75 | [M+H]^+^ | C_18_H_34_O_5_ | 329.2328 (-0.6, -1.8) | 211.1136 | Enoic acid | + |
| 43 | 8.08 | [M+H]^+^ | C_21_H_22_O_8_ | 403.1389 (-0.4, -1.0) | 373.0951 | 3,5,6,7,3’,4’-Hexamethoxyflavone | +- |
| 44 | 8.10 | [M+H]^+^ | C_42_H_62_O_17_ | 839.4064 (-0.1, -0.1) | 663.3736, 487.3416, 469.3319 | Licoricesaponin G2 | ++ |
| 45* | 8.63 | [M-H+HCOOH]^-^ | C_41_H_68_O_14_ | 829.4587 (0.1, 0.1) | 655.4437, 543.1548 | Astragaloside IV | + |
| 46* | 8.81 | [M-H+HCOOH]^-^ | C_41_H_68_O_14_ | 829.4595 (0.9, 1.1) | 785.4691, 455.3538 | Astragaloside III | + |
| 47 | 8.93 | [M+H]^+^ | C_42_H_62_O_16_ | 823.4117 (0.1, 0.1) | 647.3784, 471.3477, 453.3358 | Licoricesaponin H2 | ++ |
| 48* | 8.93 | [M+H]^+^ | C_42_H_62_O_16_ | 823.4117 (0.1, 0.1) | 647.3784, 471.3470, 453.3366 | 18*β*-Glycyrrhizic acid | ++ |
| 49* | 9.46 | [M+H]^+^ | C_21_H_22_O_8_ | 403.1391 (-0.2, -0.5) | 373.0917, 343.1187 | Nobiletin | + |
| 50 | 9.55 | [M+H]^+^ | C_42_H_62_O_17_ | 839.4067 (0.2, 0.2) | 487.3435 | Unknown | + |
| 51* | 9.74 | [M+H]^+^ | C_43_H_70_O_15_ | 827.4793 (1.0, 1.2) | 629.4058, 455.3511, 437.3423 | Astragaloside II | + |
| 52 | 9.77 | [M+H]^+^ | C_42_H_64_O_15_ | 809.4321(-0.2, -0.2) | 831.4145, 633.4026, 457.3735, 439.3582, 369.1332, 351.1234 | LicoricesaponinB2 /Dehydroxyuralsaponin C | ++ |
| 53* | 9.92 | [M+H]^+^ | C_42_H_62_O_16_ | 823.4109 (-0.7, -0.9) | 647.3786, 471.3474, 453.3362 | Licoricesaponin K2 | ++ |
| 54* | 10.18 | [M+H]^+^ | C_42_H_68_O_13_ | 781.4739 (0.1, 0.1) | 763.4631 | Saikosaponin A | + |
| 55* | 10.19 | [M+H]^+^ | C_22_H_24_O_9_ | 433.1500 (0.1, 0.2) | 418.1258, 85.0925, 373.0560 | 3,5,6,7,8,3',4'-Heptamethoxyflavone | + |
| 56* | 10.20 | [M+H]^+^ | C_42_H_62_O_16_ | 823.4116 (0, 0) | 647.3831, 471.3467, 453.3373 | 18*α*-glycyrrhizic acid | ++ |
| 57 | 10.23 | [M+H]^+^ | C_48_H_78_O_18_ | 943.5267 (0.1, 0.1) | 797.4689, 635.4105 | Soyasaponin I | + |
| 58 | 10.53 | [M+H]^+^ | C_21_H_22_O_9_ | 419.1348 (0.6, 1.4) | 371.0777 | Natsudaidain/Monohydroxy-hexamethoxyflavone | + |
| 59* | 10.63 | [M+H]^+^ | C_20_H_20_O_7_ | 373.1291 (0.4, 1.1) | 355.1839 | Tangeretin | + |
| 60 | 10.70 | [M+H]^+^ | C_42_H_68_O_13_ | 781.4730 (-0.8, -1.0) | 763.4628, 455.3517, 437.3410 | Saikosaponin B2 | + |
| 61 | 10.91 | [M+H]^+^ | C_42_H_68_O_13_ | 781.4740 (0.2, 0.3) | 763.4623, 745.4523, 455.3526, 437.3412 | Saikosaponin D | + |
| 62* | 11.27 | [M+H]^+^ | C_20_H_20_O_8_ | 389.1238 (0.2, 0.5) | 359.0757 | 5-Demethylnobiletin | + |
| 63 | 11.41 | [M+H]^+^ | C_45_H_72_O_16_ | 869.4901 (0.2, 0.2) | 851.4780, 833.4693, 689.4250, 671.4139, 455.3528, 437.3404, 143.1069 | Astragaloside I | + |
| 64 | 11.49 | [M+Na]^+^ | C_35_H_54_O_9_ | 891.4733 (1.5, 1.7) | 601.3748, 583.3643, 487.3425, 469.3308, 451.3210 | 7,8-Didehydrocimigenol-3-*O*-*β*-d-xylopyranoside | + |
| 65* | 11.58 | [M+Na]^+^ | C_35_H_56_O_9_ | 643.3825 (0.3, 0.5) | 621.4008, 603.3888, 453.3372 | Cimgenoside | + |
| 66 | 11.62 | [M+H]^+^ | C_44_H_70_O_14_ | 823.4844 (0, 0) | 805.4744, 787.4630, 455.3528, 437.3412, 419.3313 | 2''-*O*-Acetylsaikosaponin A | ++ |
| 67 | 11.95 | [M+H]^+^ | C_45_H_72_O_16_ | 869.4890 (-0.9, -1.0) | 851.4790, 833.4689, 689.4252, 671.4146, 455.3517, 437.3412, 143.1062 | Isoastragaloside I | + |
| 68 | 11.92 | [M+H]^+^ | C_21_H_22_O_9_ | 419.1339 (-0.3, -0.7) | 389.0862, 371.0766 | Monohydroxy-hexamethoxyflavone | + |
| 69 | 12.05 | [M+H]^+^ | C_44_H_70_O_14_ | 823.4846(0.2, 0.2) | 805.4732, 455.3523, 437.3420, 419.3329 | 2''-O-Acetylsaikosaponin A/B2/D | + |
| 70 | 12.22 | [M+H]^+^ | C_44_H_70_O_14_ | 823.4844 (0.0, 0.0) | 805.4724, 455.3531, 437.3405, 419.3314 | 2''-O-Acetylsaikosaponin A/B2/D | + |
| 71* | 13.81 | [M+H]^+^ | C_15_H_18_O_2_ | 231.1385 (0, 0) | 185.1330 | Atractylenolide I | + |
| 72 | 13.92 | [M+Na]^+^ | C_32_H_50_O_7_ | 569.3444 (-1.0, -1.8) | 529.3528, 511.3438, 487.3401, 469.3323 | 24-epi-7,8-Didehydrocimigenol-3-O-β-D-xylopyranoside | + |
| 73* | 14.68 | [M+H]^+^ | C_30_H_46_O_4_ | 471.3479 (0.5,1.1) | 493.3290, 53.3371, 25.3421, 407.3310, 317.2120, 271.2065 | Glycyrrhetinic acid | + |

**Note:** Rats were orally administrated with BZYQT at a dose of 20 g/kg/day for 3 consecutive days. Small intestinal contents were collected at 0.25, 0.5, 1, 2, 4 h after the last oral administration, and the samples were pretreated according to the pretreatment of feces that mentioned in our previous research of the metabolic profile of BZYQT (Hu, et al., 2019). The prototypes in the small intestinal contents were identified based on the retention time and mass fragmentation of the components characterized in BZYQT extracts (Liu, et al., 2019). *: meant that prototypes were identified with reference standards; +: MS response was greater than e^3^ and less than e^4^; ++: MS response was greater than e^4^ and less than e^5^; +++: MS response was greater than e^5^.

**Table S4.** Core signaling pathways might be related to the immunomodulatory effect of BZYQT for the treatment of poly(I:C)-induced lung inflammation

| **No.** | **Pathways** | **Count** | ***p* value** | **Genes (human)** |
| --- | --- | --- | --- | --- |
| 1 | PI3K-Akt signaling pathway | 129 | 2.38E-48 | CCND1, BCL2, BCL2L1, CASP9, CDK2, CDKN1A, CREB1, EGFR, ERBB2, FGF2, FGFR1, FLT1, FLT3, MTOR, HSP90AA1, IKBKB, IL2, IL4, **IL6**, ITGA5, ITGAV, ITGB3, JAK1, JAK2, KDR, KIT, MCL1, MYC, NFKB1, NOS3, NRAS, NTRK1, NTRK2, PDGFRA, PDGFRB, PIK3CA, PIK3CG, PIK3R1, PRKCA, MAPK1, MAPK3, MAP2K1, PTK2, RELA, RXRA, SYK, TIMP1, TLR4, TP53, VEGFA, IKBKG, CASP3, MAPK14, CTNNB1, CTSL, ESR1, HIF1A, MMP2, MMP9, PLAU, PLCG1, PTPN11, SRC, STAT3, **TNF**, PDCD4, KLK3, AR, EP300, RB1, BAX, F3, **ICAM1**, IL1B, JUN, MAPK8, **SELE**, TGFBR1, **VCAM1**, NOX4, GAPDH, HMOX1, NOS2, SLC2A1, CD81, PPARA, TNFRSF1A, ESR2, FOS, CDC25C, HDAC1, ABCB1, PTGS2, G6PD, HDAC2, PLA2G4A, PPARD, RARA, SPHK1, PGR, MMP1, BIRC3, XIAP, ADORA2A, ADORA2B, CNR1, ITGAL, TH, ADORA1, ADORA3, S1PR1, OPRM1, IRAK4, F2, PTK2B, **CXCR2**, CCNB1, CTNNA1, ALOX5, CCR1, CCR5, CCR8, CXCR4, PPARG, HLA-A, LCK, BDKRB1, PTGS1, CTSB |
| 2 | TNF signaling pathway | 94 | 3.5E-35 | BIRC3, CASP3, CASP7, CREB1, MAPK14, FOS, **ICAM1**, IKBKB, IL1B, **IL6**, JUN, MMP3, MMP9, NFKB1, PIK3CA, PIK3R1, MAPK1, MAPK3, MAPK8, MAP2K1, PTGS2, RELA, **SELE**, **TNF**, TNFRSF1A, **VCAM1**, IKBKG, NOD2, AHR, MTOR, HIF1A, HSP90AA1, IL2, IL4, JAK1, JAK2, LCK, PLCG1, RARA, RORC, RXRA, STAT3, TGFBR1, BAX, BCL2, CASP9, CTSS, EP300, HSPD1, NOS2, SRC, SYK, TLR4, VDR, SPHK1, IRAK4, TLR9, ALOX5, XIAP, BCL2L1, CCR5, PIK3CG, MMP1, MMP13, ITGB3, PPARG, CASP1, PLG, PRKCA, EGFR, FGF2, FGFR1, MYC, NRAS, NTRK1, NTRK2, PDGFRA, PDGFRB, PLA2G4A, TP53, C1R, ITGA5, CTSB, P2RX7, CYP2E1, PPARA, PTGS1, **CXCR2**, PTPN11, ADAM17, SLC2A1, NOS3, PTK2, TYMS |
| 3 | Leukocyte transendothelial migration | 18 | 5.45E-16 | MAPK14, CTNNA1, CTNNB1, PTK2B, **ICAM1**, ITGAL, MMP2, MMP9, PIK3CA, PIK3R1, PLCG1, PRKCA, PTK2, PTPN11, **VCAM1**, CXCR4, ITGA5, SRC |
| 4 | Cytokine-cytokine receptor interaction | 24 | 3.23E-14 | CCR1, CCR5, CCR8, EGFR, FLT1, FLT3, IL1B, IL2, IL4, **IL6**, **CXCR2**, KDR, KIT, PDGFRA, PDGFRB, TGFBR1, **TNF**, TNFRSF1A, VEGFA, CXCR4, ADRB2, HLA-A, NTRK1, SRC |

Note: The bold font genes marked with blue were validated through experiments.

**References**

Hu, L., Yao, Z., Qin, Z., Liu, L., Song, X., Dai, Y., Kiyohara, H., Yamada, H., Yao, X., (2019). *In vivo* metabolic profiles of Bu-Zhong-Yi-Qi-Tang, a famous traditional chinese medicine prescription, in rats by ultra-high-performance liquid chromatography coupled with quadrupole time-of-flight tandem mass spectrometry. *J. Pharm. Biomed. Anal.* 171, 81-98. 10.1016/j.jpba.2019.04.001.

Liu, L., Hu, L., Yao, Z., Qin, Z., Idehara, M., Dai, Y., Kiyohara, H., Yamada, H., Yao, X., (2019). Mucosal immunomodulatory evaluation and chemical profile elucidation of a classical traditional chinese formula, Bu-Zhong-Yi-Qi-Tang. *J. Ethnopharmacol.* 228, 188-199. 10.1016/j.jep.2018.08.003.
